# Supplementary material for: Extended Spectrum β-Lactamase-Producing Enterobacterales of Shrimp and Salmon Available for Purchase by Consumers in Canada—A Risk Profile Using the Codex Framework
Source: Antibiotics (Basel). 2023 Sep 6;12(9):1412. doi: 10.3390/antibiotics12091412 (PMC10525137; doi:10.3390/antibiotics12091412)
Supplement: Supplementary file 1 [file antibiotics-12-01412-s001.zip › Supplementary Material_ESBLRP_fnl proof.pdf]

Antibiotics – Supplementary Materials

Extended Spectrum β-Lactamase-Producing Enterobacterales of Shrimp and Salmon Available for Purchase by Consumers in Canada—A Risk Profile Using the Codex Framework

F. Carl Uhland [1,*], Xian-Zhi Li [2], Michael R. Mulvey [3], Richard Reid-Smith [1], Lauren M. Sherk [1], Hilary Ziraldo [1], Grace Jin [1], Kaitlin M. Young [3], Mark Reist [2] and Carolee A. Carson [1]

[1] Centre for Foodborne, Environmental and Zoonotic Infectious Diseases, Public Health Agency of Canada, Guelph, ON N1H 7M7, Canada
[2] Veterinary Drugs Directorate, Health Products and Food Branch, Health Canada, Ottawa, ON K1A 0K9, Canada
[3] National Microbiology Laboratory, Public Health Agency of Canada, Winnipeg, MB R3E 3R2, Canada;
* Correspondence: frederick.uhland@phac-aspc.gc.ca

Supplementary Materials

Table S1. Summary of data quality, level of concern, data gaps

Sections		Data available (Y/N/S/L[#])	New Data Needed to Make Risk Management Decision?
1. Description of the AMR food safety issue (per Codex definition)		N/A	
2. Information on the AMR organism /determinant		Data Quality Score summary: 5.9; Level of Concern summary: 3	
2.1. Characteristics of ESBL-EB	2.1.1. Sources and transmission routes	S	Yes<br>Surveillance of retail seafood in Canada, limited to pilot projects
	2.1.2. Pathogenicity, virulence, and linkages to resistance of particular strains	S	Yes<br>More data are needed on the genetic linkages of pathogenicity, resistance, and virulence characteristics, and its impacts on human illness. Information from whole genome sequencing (WGS) would be beneficial.
	2.1.3. Growth and survivability, including inactivation in foods (D-value, minimum pH for growth) of *E. coli* in seafood to fork continuum	Y	No<br>Guidelines for bacterial growth and survivability in seafood are published.
	2.1.4. Distribution, frequency and concentrations in the food chain	L	Yes<br>Yes, more data needed on *E. coli* and resistance prevalence/concentrations within shrimp and salmon products imported and produced in Canada to quantify the risk posed by the products to Canadians.
2.2. Characteristics of ESBL-EB isolated from retail shrimp and salmon	2.2.1. Resistance mechanisms and location of the resistance determinants	Y	Yes<br>ESBL resistance and associated ARGs are well characterized. However, localization (MGE) and co-localization with other ARGs is of concern. Information from WGS would be beneficial.
	2.2.2. Cross-resistance and/or co-resistance to other antimicrobial agents	Y	Yes<br>Even though phenotypic data exist in Canada, knowledge of the genes involved in cross-

Sections		Data available (Y/N/S/L#)	New Data Needed to Make Risk Management Decision?
			resistance/co-selection would assist decision making about interventions. Information from WGS would be beneficial.
	2.2.3. Transferability of resistance determinants between microorganisms	Y	Yes<br>Antimicrobial resistance gene (ARG) transfer has been proven, however more information could be gathered about the rate of transfer. WGS would be beneficial in investigating this aspect.
3. Information on the antimicrobial agent(s) to which resistance is expressed – third generation cephalosporins		Data Quality Score summary: 8.0; Level of Concern summary: 3	
3.1. Class of the antimicrobial agent(s)		Y	No
3.2. Non-human uses of third generation cephalosporins.	third generation cephalosporins are not reported as being used in aquaculture	S	Yes. Continued surveillance of the literature should be undertaken to identify changes in usage practices
3.3 Human uses of third generation cephalosporins.	3.3.1 Spectrum of activity and indications for treatment	Y	No
	3.3.2 Importance of the antimicrobial agent, including consideration of critically important antimicrobial lists	Y	No
	3.3.3 Distribution, cost and availability	Y	No
	3.3.4 Availability of alternative antimicrobial agent(s)	Y	No
	3.3.5 Trends in the use of the antimicrobial agent(s) in humans and information on emerging diseases due to microorganism(s) resistant to the antimicrobial agent(s) or classes.	Y	No
4. Information on the Food Commodity – salmon and shrimp		Data Quality Score summary: 6.9; Level of Concern summary: 2.5	
4.1 Source(s) (domestic or imported), production volume, distribution and per capita consumption of foods or raw materials identified with the AMR hazard(s) of concern.		Y	Yearly Canadian production data are available. Surveys of per capita consumption are repeated regularly (e.g., Foodbook 2.0)
	4.1.1 Characteristics of the food product(s) that may impact risk management (e.g., further processed, consumed cooked, pH, water activity, etc.)	Y	Unlike other types of animals products (e.g., poultry), seafood may consumed raw (e.g., Sushi), making growing, processing and retail contamination concerning. Lacking information on different forms

Sections		Data available (Y/N/S/L#)	New Data Needed to Make Risk Management Decision?
			associated with higher probabilities of foodborne infection, or impact risk management.
	4.1.2 Description of the food production to consumption continuum (e.g., primary production, processing, storage, handling, distribution and consumption) and the risk factors that affect the microbiological safety of the food product of concern.	Y	Yes<br>The majority of the shrimp consumed in Canada are imported. Regulation differences concerning AMU, hygiene etc. need to be considered in any analysis of risk. There are many opportunities throughout the food production to consumption continuum for contamination to occur. Would benefit from Canadian-specific quantitative studies.
5. Information on adverse public health effects		Data Quality Score: 6.2; Level of Concern: 3	
5.1 Characteristics of the disease caused by the identified foodborne AMR microorganisms or by pathogens that have acquired resistance determinants via food	5.1.1. Trends prevalence and nature of AMR foodborne disease in people	Y	Yes<br>Surveillance of ESBL-EB infections in Canada is currently undertaken by CANWARD and the Canadian Nosocomial Infection Surveillance Program (CNISP). Trends are increasing; Up to date Canadian passive surveillance data are available but there are data gaps with the burden of illness. Additionally, cases attributed to food consumption are unknown. WGS analysis could provide information concerning source attribution of ESBL-EB isolates.
	5.1.2. Epidemiological pattern (outbreak or sporadic) regional, seasonal or ethnic differences in the incidence	S	Yes<br>Epidemiological patterns thus far identified are associated primarily with hospital care, where

Sections		Data available (Y/N/S/L[#])	New Data Needed to Make Risk Management Decision?
			surveillance activities are concentrated. Surveillance of food animals has been undertaken by CIPARS but salmon, shrimp and seafood are not routinely included. Global dissemination of ESBL-EB is of concern.
	5.1.3. Susceptible population and risk factors	L	Yes<br>Information concerning risk factors and susceptible populations is unknown. Consideration of populations at risk, which may consume seafood raw.
	5.1.4. Regional, seasonal and ethnic differences in the incidence of foodborne disease due to AMR hazard(s)	S	Yes<br>Information concerning risk factors and susceptible populations is unknown. Consider here ethnic populations which consume seafood raw. Travel associated ESBL carriage has also been demonstrated.
	5.1.5. Consequences of AMR on the outcome of the disease Burden of illness (BOI)	N	Yes<br>Information examining BOI of ESBL-EB infections is limited. Given the available information, it is reasonable to expect increases in mortality and hospital costs, encompassing increased LOS and treatment failures, from ESBL-EB, compared to infections caused by susceptible bacteria.
6. Risk management options		Data Quality Score: 5.6; Level of Concern: 3	
6.1. Identification of risk management options to control the AMR hazard along the production to consumption continuum both in the pre-harvest and post-harvest stages	6.1.1. Measures to reduce the risk related to the selection and dissemination of foodborne AMR microorganisms(s) (summarized from Loest et al. 2022)	L	Farm level data is difficult to obtain from importing countries, and instigating change from importers of seafood to Canada would be difficult. Risk reduction in imported seafood products would likely target processing and retail sectors where governmental and hygienic controls (HAACP) are in place.
	6.1.2. Measures to minimize the contamination and cross-contamination of food by AMR microorganism(s) (summarized from Loest et al. 2022)	S	Several studies have demonstrated the utility of HAACP programs in seafood processing plants. A qualitative description of risk management options is available; quantitative data supporting the effectiveness of these measures is lacking.

Sections		Data available (Y/N/S/L[#])	New Data Needed to Make Risk Management Decision?
	6.1.3 Utilization of Whole Genome Sequencing as a surveillance-based risk management tool to control the AMR hazard along the sea-food to fork continuum	L	WGS can be valuable in determining the impact of farming practices on AMR, virulence, and survival. It would also be useful for source attribution and control in the processing and distribution chain

N/A – not applicable; *Data quality score: data were only scored as it pertains to risk, background information were not scored; #: Y-Yes, N-No, S-Some, L-Limited

Table S2. Growth limiting temperature, pH, NaCl%, water activity and thermal resistance values for select Enterobacterales

**Pathogenic bacteria**	**Temperature °C**	**pH**	**NaCl%**	**Water Activity (A_w)**	**Thermal Resistance**
	Min/Max	Min/Max	Maximum	Minimum	Ave. D_{10} @ 55°C/65°C in seconds (for catfish and tilapia)
Salmonella	5.2-46.2	3.7/9.5	4–5	0.94	417.5/2.0
Shigella	6.1-47.1	4.8/9.3	4–5	0.96	-
Pathogenic *E. coli*	6.5-49.4	4.0/10.0	6	0.95	493/3.75

Adapted from [51, 55, 257].

Table S3. Enterobacterales described in shrimp aquaculture, region of detection and concentration/prevalence in the source

Country	Bacteria	Source water (concentration or prevalence (%))	Culture water (concentration or prevalence (%))	Cultured shrimp	Wild shrimp	Reference
Indonesia	*Enterobacteriaceae*<br>*E. coli*	-	<1- 4.0 log_{10} CFU/g<br><1-4.0 log_{10} CFU/g	<1- 5.7 log_{10} CFU/g<br><1-4.0 log_{10} CFU/g	<1- 6.8 Log_{10} CFU/g<br><1 -3.8 log_{10} CFU/g	[80]
Benin	*Enterobacteriaceae*	-	-	-	1.4 -3.2 log CFU/g	[258]
Six countries (non identified)	Fecal coliforms; *Salmonella*	42.86%@>1000cells/ 100 ml; 8.2%	4.76%@>1000cells/100ml; 3.5%	12.15%@>1000cells/ 100ml;1.6%	-	[16]
India	Total coliforms	2.48 ± 0.14 (log_{10} MPN/g)	3.22 ± 0.72 (log_{10} MPN/g)	3.28 ± 0.32(log_{10} MPN/g)	-	[70]
India	Fecal coliforms	-	-	-	MPN 9.5/g	[259]
Bangladesh	*Salmonella*<br>*E. coli*		7/16 (43.7%)<br>10/16 (62.5%)	6/30 (20%)<br>18/30 (60.5%)	-	[71]
Bangladesh	Total coliform	-	2.07-2.32 (Mean microbial counts - log10 CFU/g)	1.96-2.46 (Mean microbial counts - log10 CFU/g)	-	[260]
Nigeria	*E. coli*<br>*Proteus vulgaris*<br>*Shigella* spp.<br>*Salmonella*<br>*C. freundii*	-	-	-	*M. vollenhovenii*/ *P. atlantica* (10^4 CFU/ml)<br>5.56/3.35<br>7.18/7.58, 7.64/5.04<br>3.64/1.04<br>7.64/1.10	[261]
Iran	*Salmonella*	-	-	-	1.8%	[262]
Thailand	Enterobacterales (*E. coli*, *Klebsiella*, *Enterobacter*)		29/87 fish and shrimp ponds (33%)			[263]
Bangladesh	*E. coli*	-	8.4-14.4 (n=NRa)	2.7-3.9% (n=NR)	-	[264]

Country	Bacteria	Source water (concentration or prevalence (%))	Culture water (concentration or prevalence (%))	Cultured shrimp	Wild shrimp	Reference
			408-1034 CFU/ml	189-196 CFU/g		

Table S4. Contamination of Enterobacterales described in the shrimp products at processing plants, region of detection and concentration/prevalence in the source

Country/bacteria	Sample	Bacteria	Prevalence (%)/Concentration	Reference
Egypt	Shrimp; frozen block and individual quick freezing (IQF) products	*Enterobacteriaceae*	25%-90%<br>Mean counts 1.5 x 10 – 1.5x103 CFU/g	[79]
Bangladesh	Processing plant; shrimp, baskets, mats	*Salmonella*<br><br>*E. coli*	9/45 (20%), 9/16(56.3%), 3/13 (23.1%).<br><br>24/45 (53.3%), 9/16 (37.5%) and 12/13 (92.3%)	[71]
India	Processing plants; prawn	*Salmonella*<br>*E. coli*	42% (headless prawn, n=12)<br>50% (whole prawns, n=6)<br>25% (headless prawns, n=12)	[160]
India	Processing plant; different IQF products, domestic source	 Coliforms<br>*Salmonella*<br>*E. coli*	(n=2210)<br>3.8-25.8%<br>0-0.1%<br>1.3-4.8%	[265]
India	Processing plant; raw and cooked IQF shrimp products	Coliforms (100/1000 counts/g)<br><br>*Salmonella*<br>*E. coli*	12%/3% (raw)<br>3%/0.1%(cooked)<br>0.1%(raw shrimp)<br>0.1% (n=1264 – raw shrimp)<br>2% (n=1264 – raw shrimp)	[266]
Indonesia	Processing plant; shrimp, various phases of processing	*Enterobacteriaceae*<br>*E. coli* (shrimp)	log10^3-10^5 CFU/g<br>0-2.9 log10 CFU/g	[80]
India	Processing plant, shrimp<br>Landing Center, shrimp	*E. coli*<br>*E. coli*	8.6% (n=23)<br>16% (n=25)	[267]
Nigeria	Processing	 *Proteus vulgaris.* (boiled shrimp)<br>*Shigella* spp. (boiled shrimp)<br>*E. coli* (sun-dried shrimp)	*M. vollenhovenii*/*P. atlantica* (10^4 CFU/ml)<br>1.78/0.17<br>1.86/2.33<br>3.11/0.45	[261]

Table S5. Enterobacterales described in shrimp and salmon at retail, region of detection and concentration/prevalence in the source

Country/bacteria	Sample	Bacteria	Prevalence (%)/Concentration	Reference
England (source countries not specified)	Retail Shrimp	*E. coli*	Whole - 2 (n=148) @1.0-1.99 log_{10} CFU/g Peeled – 1 (n=148) @2.0-2.99 log_{10} CFU/g	[268]
Bangladesh	Retail Shrimp (various tissues)	*E. coli* Fecal coliforms *Klebsiella*	1.2×10^3 - 1.2×10^5 CFU/g 1.2×10^4 (eggs) – 2.0×10^6 2.0×10^3 (eggs) – 9.0×10^5	[269]
Brazil	Retail Shrimp (domestic source)	*E. coli*	33.7-40% (n=30) Range 10^0->10^4 CFU/g	[270]
India	Retail	*Salmonella* (*S.* Typhi, *S.* Typhimurium, *S.* Paratyphi B, *S.* Senftenberg, and *S.* Senftenberg, *S.* Weltevredenand)	36/237 (15%)	[84]
India	Retail shrimp	*E. coli*	15% (n=20)	[267)
Indonesia	Industry/Grocery store; frozen shrimp	*Enterobacteriaceae* *E. coli*	3-5.3 log10 CFU/g 1-3.5 log10 CFU/g	[80]
India	Fish harbors, fish markets fish/shrimp	*Salmonella* (Mbandaka, Derby, *Salmonella* VI, Braenderup, Weltevreden and Rissen were the most prevalent)	Shrimp 23/86 (23%)	[86]
USA	Grocery stores (imported) salmon (n=63) shrimp (n=38)	*Salmonella* (salmon/shrimp) *Shigella* (salmon/shrimp) *E. coli* (salmon/shrimp)	14.3%/21.1% 36.5%/28.8% 4.8%/10.5%	[249]
Egypt	Retail	*Salmonella* (Typhimurium, Derby, Typhi, Paratyphi A, and Abortus equi)	7/50 (14%)	[85]
Brazil	Grocery stores	Total coliforms	< 3.0 - 1.1×10^3 MPN/g	[271]

	Salmon (n=31)	Thermotolerant coliforms	< 3.0 - 4.6 x 10^2 MPN/g	
USA	Retail<br>Salmon	*E. coli*	1.5% (combined)<br>(internet source, n=34)<br>(local source, n=32)	[272]
Brazil	Retail (farmed shrimp)<br>- 5 pooled 500 g samples	*E. cloacae*<br>*K. pneumoniae* and *E. coli*<br>*C. freundii* and *E. aerogenes*	5/5<br>4/5<br>1/5	[273]
Czech Republic (source countries not specified)	Retail<br>Salmon	*E. coli*	$3.5x10^1$-$4.5x10^4$ CFU/g<br>(1.6-1.7 $\log_{10}$ CFU/g)	[52]
Germany	Retail<br>Salmon<br>Shrimp	*E. coli*	Shrimp (n=16)<br>31.3% (fresh)<br>2.59 log CFU/g;<br>12.5% 9 (frozen)<br>2.7 log CFU/g.<br><br>Salmon (n=21)<br>0-23.8% (fresh)<br><2.0-2.2 log CFU/g<br>0-4.8 % (frozen)<br><2.0-2.3 log CFU/g	[274]
India (source countries not specified)	Retail<br>Shrimp	*E. coli*	2% (n=50)	[275]
Brazil	Retail<br>Salmon	*E. coli*	<3.0-4.6x102 MPN/g<br>(thermotolerant coliforms)<br>(n=31)	[271]
India	Retail<br>Shrimp	*E. coli*<br>Sample 1; n=60<br>Sample 2; n=40	 40%<br>48.3%	[276]

CFU, colony forming unit; MPN, most probable number.

Table S6. ESBL-EB in the aquaculture continuum

**Country**	**Product/Sample**	**Bacteria**	**Prevalence of ESBL**	***bla* genes**	**Reference**
USA	Ready to eat shrimp	*E. coli*	2 isolates resistant to ceftriaxone identified in 8 samples	Phenotypic ID resistance to ceftriaxone	[277]
India	Retail seafood<br>19 samples, which included 14 finfish and five shellfish samples	*E. coli*	1/14 finfish	bla_{SHV}, bla_{TEM}, $bla_{CTM-X-1}$, $bla_{CTM-X-25}$, bla_{OXA-1}, bla_{NDM-5}	[278]
China	Farmed fish/gut samples	*E. coli*	3/218 (1.5%)	$bla_{CTX-M-14}$, $bla_{CTX-M-79}$, bla_{SHV-27}	[279]
Singapore	Culture waters	*E. coli*	27%	bla_{CTX-M}, bla_{SHV}	[280]
Vietnam	Red tilapia, striped catfish, wild fish	*E. coli*	38/106 (35·9%)	bla_{CTX-M}, bla_{TEM}	[75]
Egypt	Tilapia/environment	*E. coli,*<br>*K. pneumoniae*<br>*Enterobacter* spp.	15/30 (50%)-	$bla_{CTX-M-15}$, bla_{SHV}, bla_{TEM}, bla_{PER-1}	[68]
Tanzania	Tilapia/poultry integrated system **T**ilapia (n=105)<br>**I**nlet **W**ater (n=30)<br>**O**utlet **W**ater (n=26)	**T**; *E. coli; (45/105)*<br>*E. cloacae; (12/105)*<br>*K. pneumoniae; (9/105)*<br>**IW**; *E. coli; (9/30)*<br>*E. cloacae; (4/30)*<br>*K. pneumoniae; (2/30)*<br>**OW**; *E. coli; (12/26)*<br>*E. cloacae; (2/26)*<br>*K. pneumoniae; (2/26)*	***E. coli***<br>T- 36%<br>IW-67%<br>OW-58%<br>***E. cloacae***<br>T- 75%<br>IW-33%<br>OW-50%<br>***K. pneumoniae***<br>T- 57%<br>IW-0%<br>OW-50%	bla_{CTX-M}, bla_{SHV}, bla_{TEM}	[67]

Germany	Retail<br>Venus clam	*E. coli*	1 isolate from 45 screened Enterobacteriaceae	*aac*A_4-like, *aad*A_1, *aph*$_{(3')}$-XV, *bla*$_{ACC-1}$, *bla*$_{SHV-12}$, *bla*$_{VIM-1}$, *cat*B_2, *dfr*A_{14}-like, *mph*(A), *qnrS*$_1$, *str*A-like, *str*B-like, *sul*$_1$, *sul*$_2$	[45]
India	Shrimp/ sediment, water, shrimp	*E. coli*<br><br><br><br>*K. pneumoniae*	CTX-M1/CTX-M9<br>sediment (15%/3%)<br>water (3%/0%)<br>shrimp (8%/8%)<br>CTX-M-1<br>water (5%)<br>shrimp (13%)	*bla*$_{CTX-M-1}$, *bla*$_{CTX-M-9}$	[7]
Cambodia	Retail fish	 <br>*S. enterica;*<br><br>*E. coli;*	ESBL+<br><br>10/60 (17%)<br><br>32/60 (53%)	 *bla*$_{CTX-M-55,}$ *bla*$_{CTX-M-130}$<br>*bla*$_{CTX-M}$	[97]
Germany	Retail seafood	*Enterobacteriaceae* (AmpC producing and ESBL+)	Prevalence in shrimp 18/80 (22.5%)<br>Prevalence in bivalves 16/80 (20%)	*bla*$_{SHV}$, *bla*$_{ACC}$, bla$_{CTX-M}$, *bla*$_{TEM}$, *bla*$_{CMY-2}$, *bla*$_{DHA}$	[103]

Table S7. Burden of illness summary for antimicrobial-resistant Enterobacterales

**Bacteria**<br>**Year (Location)**	**Study Type**	**Burden of illness measures**	**Summary of Findings**	**Reference**
Evaluation of the health and healthcare system burden due to antimicrobial-resistant *E. coli* infections in humans, studies published after December 31, 1998.	Systematic review of the literature	30 day and all cause mortality	There was a significant 30-day and all-cause mortality burden associated with *E. coli* infections.	[198]
Estimating the burden of antimicrobial resistance, studies published between Jan 2013-Dec 2015	Systematic review of the literature	Patient or provider/payer burden<br>Mortality<br>Healthcare system costs	There was substantial variability in burden estimates.<br>This may lead to inaccurate intervention evaluations and poor policy/investment decisions.	[199]
ESBL-EB infections after rectal colonization with ESBL-producing *E. coli* or *K. pneumoniae*, 2014-2015 (Germany)	Prospective cohort	Disease outcome<br>Hospital length of stay (LOS)<br>Mortality	More severe medical conditions were associated with ESBL-KP as compared to ESBL-EC<br>LOS (days); ESBL-EC/ESBL-KP, 11 (6–22)/16 (7–36), p<0.001<br>Mortality; ESBL-EC/ESBL-KP, 4.2%/6.7%, p=0.013	[201]
Sepsis Caused by ESBL-Positive *K. pneumoniae* and *E. coli*, 2008-2011 (Germany)	Retrospective cohort	Mortality<br>Severity of illness	No significant difference in mortality was noted between patients with ESBL-KP-Bac compared to ESBL-EC-Bac (27.1% vs. 23.8%). ESBL-KP-Bac infections were associated with greater disease severity than ESBL-EEC-Bac infections	[200]
Spontaneous bacterial peritonitis, 2003-2011(Canada)	Retrospective cohort	30 day mortality	Resistance to 3GCs was a positive predictor for 30 day mortality (OR 5.3 [1.3 to 22])	[210]
ESBL-producing *E. coli* 2007 (UK)	Cohort	Mortality	Significantly higher proportion of patients with ESBL-producing *E. coli* died compared to those with non-ESBL producing *E. coli* (Adjusted OR 3.57; 95%CI 1.48-8.60; p<0.005).	[204]

ESBL-producing *E. coli*<br>1997-2005 (Spain)	Retrospective Cohort	Mortality	Morality was higher in patients with bacteremia due to ESBL-producing *E. coli* infections (RR 3.64; 95%CI 1.41-9.4; p=0.03).	[205]
Neutropenic cancer patients with ESBL-*E. coli* bacteremia<br>2006-2008 (Spain)	Prospective Observational Case-control	ICU Admission, Mortality	Neutropenic patients with ESBL-EC bacteremia presented with increased ICU admission (25% compared to 4%; p=0.04) and higher overall mortality rate (37.5% compared to 6.5%; p=0.01) compared to the susceptible control group.	[202]
Bacteremia caused by *E. coli*<br>200-2007 (Italy)	Retrospective cohort	30-day mortality	ESBL production independently associated with 30-day mortality among patients with hematological malignancies and bloodstream infections caused by *E. coli* (OR 8.84; 95%CI 1.48-52.91; p=0.01).	[208]
Enterobacteriaceae bacteremia<br>2006 (Israel, USA)	Systematic review and Meta-analysis	Mortality and delay in effective therapy	Increased mortality in ESBL-producing *Enterobacteriaceae* bacteremia compared to non-ESBL producing *Enterobacteriaceae* bacteremia (Pooled RR 1.85; 95%CI 1.39-2.47; p<0.001).<br>Significant association between ESBL production and delay in effective therapy compared to non-ESBL associated Enterobacteriaceae bacteremia (pooled crude RR 5.56; 95%CI 2.94-10.51; p<0.001).	[207]
Enterobacteriaceae causing bacteremia<br>2010 (Netherlands)	Systematic review and meta-analysis	Mortality	ESBL-producing *Enterobacteriaceae* bacteremia associated with increased mortality compared to non-ESBL *Enterobacteriaceae* bacteremia. (Pooled OR 2.35; 95%CI 1.9-2.91)	[206]
Hospital patients with ESBL-producing *E. coli* or *Klebsiella* spp. infections other than UTIs.<br>2001-2004 (USA)	Matched-cohort	Hospital cost, LOS	Total costs significantly greater for patients with ESBL-producing *E. coli* or *Klebsiella* spp. compared to non-ESBL. Mean additional cost of $16,451 per patient (95%CI $965-$31,937). Length of stay was 9.7 days longer for ESBL patients (95%CI 3.2-14.6 days; p=0.006).	[214]
Patients hospitalized with UTI caused by ESBL *E. coli* and *Klebsiella* spp. (ESBL-EK)<br>2011-2012 (USA)	Retrospective, matched-cohort	LOS, hospital costs	23.6% of ESBL-EK patients received appropriate initial antibiotic therapy, compared to 98.2% of non-ESBL patients (p<0.001).<br>Median LOS significantly longer for ESBL patients compared to non-ESBL patients (6 days compared to 4 days, p=0.02)<br>Hospital costs significantly higher for ESBL patients compared to Non-ESBL patients ($10,741 compared to $7,083 p=0.02).	[215]

ESBL *E. coli* Bloodstream infection<br>2006 (Italy)	Retrospective cohort	LOS, hospital costs, 21-day mortality rate<br>Treatment failure rate after 72hrs	ESBL BSIs associated with longer (+7 days) and more expensive (+5,026EUR) hospital stays compared to non-ESBL BSIs.<br>21-day mortality higher among patients with ESBL BSIs compared to non-ESBL BSIs. (OR=4.43; 95%CI=2.46-7.95; p<0.001).<br>Treatment failure rates after 72hrs higher among pts with ESBL BSIs compared to non-ESBL BSIs (OR=3.21; 95%CI=1.96-5.27; p<0.001).	[209]
ESBL-producing *E. coli* or *Klebsiella* spp. (ESBL-EcKs)<br>2010-2013 (Canada)	Retrospective case-control	Clinical outcome, mortality, hospital cost	Hospital cost higher in ESBL-EcKs cases compared to non-ESBL-EcKS controls (median additional cost $3,416; p=0.04). Costs primarily due to hospital stay (8 vs 6 days; p=0.02), and location (ward vs ICU).<br>Clinical failure more likely in ESBL-EcKs cases compared to Non-ESBL-EcKs controls (RR=2.38; 95%CI=1.11-5.09; p=0.03).<br>All-cause mortality higher in ESBL-EcKs cases compared to non-ESBL-EcKs controls (RR=3.25; 95%CI=1.11-9.51; p=0.04).<br>Adequate initial antimicrobial treatment received in 96% of non-ESBL-EcKs controls, compared to 48% of ESBL-EcKs cases (p<0.01).	[203]
Infection due to ESBL-producing *E. coli* or *K. pneumoniae*<br>1997-1998 (USA)	Retrospective cohort	Clinical outcome, microbiological outcome, mortality, duration of hospital stay, hospital charges	ESBL-EcKp cases associated with higher median hospital costs compared to non-ESBL-EcKp controls (2.90 times higher cost; 95%CI 1.76-4.78; P<0.001). ESBL-EcKp cases demonstrated increased median hospital LOS compared to non-ESBL-EcKp controls (1.76 times longer; 95%CI 1.17-2.64; p=0.01).	[194]
ESBL and AmpC *E. coli* or *Klebsiella* spp. inpatient hospital infections<br>2004 (Canada)	Case-control	Appropriate use of antibiotics<br>Number of days to appropriate antibiotics	Non-ESBL/AmpC Controls were more likely to be given appropriate antibiotics compared to ESBL/AmpC cases (27% vs 13.8%; p=0.05).<br>ESBL/AmpC Cases had longer number of days to appropriate antibiotics compared to non- ESBL/AmpC controls (median 2.8 days vs 1.2 days; p=0.05).	[197]
Modeling of extra length of stay due to nosocomial infection, 2011 (Argentina)	Multistate model that accounted for	LOS	Ignoring time dependance (time of infection) overestimates the extra length of stay	[211]

	the time of infection.			
BSI due to ESBL-positive *K. pneumoniae* and *E. coli*, 2008-2011 (Germany)	Retrospective cohort	Increased hospital costs<br>LOS	ESBL-E^{+}, BSI cases; median total LOS of 30 days (IQR 14–60), ESBL-E^{-}, BSI cases total LOS 16 days (IQR 9–37; p < 0.001<br>Increased LOS and hospital costs were associated with ESBL+ *K. pneumoniae*, when compared to *E. coli*.	[213]
Health care associated infection (HAI), not specifically ESBL, 2018-2019 (UK)	One-year prospective incidence study	LOS	HAI caused an increase of LOS by 7.8 days (95% confidence interval (CI): 5.7–9.9).	[212]
Infections caused by ESBL-producing *E. coli* and *K. pneumoniae*, 2002-2004 (Germany)	Case-control	Cost of treatment<br>Duration of hospitalization<br>Hospital mortality	Respiratory tract and bloodstream infections incurred higher medication costs (325 vs 58.9 Euros, p=0.002) and longer hospital stays (50% vs 6.67%, p=0.034).<br>In hospital mortality was not affected by the present of ESBL	[216]
ESBL producing *E. coli*, 2021 (Canada)	Scoping review	-	Summarize the characteristics of BOI reporting in the ESBL-EC literature	[217]
Community-associated ESBL-*E. coli* acute pyelonephritis (APN), 2007-2013 (Republic of Korea)	Case-control	Severity of illness<br>Clinical cures<br>Microbiological cures	ESBL productions was not a significant factor causing microbiological/clinical failure (hazard ratio HR, 0.49; 95% CI, 0.21 to 1.12; P = 0.091)/[HR], 0.39; 95% CI, 0.12 to 1.30; P = 0.126)	[219]
Expert Panel on the Potential Socio-Economic Impacts of Antimicrobial Resistance in Canada, 2019 (Canada)	Review	-	Hospital Costs of Antimicrobial Resistance in Canada<br>The Impact of Antimicrobial Resistance on the Canadian Economy<br>The Social Impacts of Antimicrobial Resistance	[2]
Adults with UTI caused by ESBL-producing *E. coli*, 2010-2013 (Spain)	Matched cohort	Clinical failure<br>Economic impact: hospital costs, treatment costs	Clinical failure in the first 7 days associated with prior AMU (p = 0.007) and ESBL presence (p < 0.001);<br>Pharmacy and hospital expenses were higher with ESBL infections as compared to non-ESBL (p < 0.001); Higher costs associated with UTI were significantly associated with ESBL production (p = 0.008)	[218]
